# A New Strategy for Achieving Shape Memory Effects in 4D Printed Two-Layer Composite Structures

**DOI:** 10.3390/polym14245446

**Published:** 2022-12-13

**Authors:** Davood Rahmatabadi, Mohammad Aberoumand, Kianoosh Soltanmohammadi, Elyas Soleyman, Ismaeil Ghasemi, Majid Baniassadi, Karen Abrinia, Ali Zolfagharian, Mahdi Bodaghi, Mostafa Baghani

**Affiliations:** 1 School of Mechanical Engineering, University of Tehran, Tehran 14174, Iran; 2 Faculty of Processing, Iran Polymer and Petrochemical Institute, Tehran 14975, Iran; 3 School of Engineering, Deakin University, Geelong 3216, Australia; 4 Department of Engineering, School of Science and Technology, Nottingham Trent University, Nottingham NG11 8NS, UK

**Keywords:** shape memory effect, fused deposition modeling, 4D printing, two-layer composite structures, shape recovery

## Abstract

In this study, a new strategy and design for achieving a shape memory effect (SME) and 4D printed two-layer composite structures is unveiled, thanks to fused deposition modeling (FDM) biomaterial printing of commercial filaments, which do not have an SME. We used ABS and PCL as two well-known thermoplastics, and TPU as elastomer filaments that were printed in a two-layer structure. The thermoplastic layer plays the role of constraint for the elastomeric layer. A rubber-to-glass transition of the thermoplastic layer acts as a switching phenomenon that provides the capability of stabilizing the temporary shape, as well as storing the deformation stress for the subsequent recovery of the permanent shape by phase changing the thermoplastic layer in the opposite direction. The results show that ABS–TPU had fixity and recovery ratios above 90%. The PCL–TPU composite structure also demonstrated complete recovery, but its fixity was 77.42%. The difference in the SME of the two composite structures is related to the transition for each thermoplastic and programming temperature. Additionally, in the early cycles, the shape-memory performance decreased, and in the fourth and fifth cycles, it almost stabilized. The scanning electron microscopy (SEM) photographs illustrated superior interfacial bonding and part integrity in the case of multi-material 3D printing.

## 1. Introduction

In the last two decades, 3D printing or additive manufacturing (AM) has been highly regarded by researchers, and this attention has augmented day by day, leading to the invention and development of different methods to employ a wider range of materials and applications [1,2,3]. The pervasiveness of AM methods, reduction of consumables and auxiliary equipment, and elimination of the constraints of manufacturing complex geometries as the main advantage of 3D printing lead to increased attention to nature-inspired structures to remove dynamic limitations of the performance in special applications, such as medicine and soft robotics [2,4]. Shape memory materials are a group of smart materials that can memorize their original form and retrieve it using the appropriate stimulus. These materials can be classified into three main categories: polymers, alloys, and hydrogels. Shape memory polymers (SMPs) have advantages due to their cheapness, simple processing, multiple stimuli, biocompatibility, high deformation, and control properties [5,6]. Few pure polymers have the SME, and polymerization methods, copolymers, and blended polymers are often used to achieve an SME because polymers must have two parts: a rigid or hard segment and an elastic or soft segment, which create a constraint and store force, respectively [7,8,9]. Most AM methods can be used for 4D printing, but they are not suitable in every field and have challenges. In addition to the challenges of smart material processing, the addition of new printing material feed-stock issues have led to many limitations in 4D printing. FDM is known as the simplest, cheapest, and most widely used method [10,11,12]. Four-dimensional printing with FDM can be achieved in a variety of ways, which are generally different from the usual method, in which the existence of smart material is necessary [13]. Indeed, the smart materials used in FDM are limited to a few thermoplastics, such as PU-based SMPs, PLA, PETG, and a few blends that are challenging to print [5,14,15,16,17,18,19,20]. In other words, 4D printing of a smart structure using FDM technology can be conducted regardless of the need for programming, as well as the smart characteristics of the printing material [21]. The SME programming procedure involves a thermomechanical cycle that is associated with applying strain at high temperatures (heating and loading) and with storing at low temperatures (unloading and fixing) [10]. These steps can be done continuously during the extrusion and deposition stages of printing by the FDM process, which is called in-printing programming [22,23]. The nature of extrusion in the FDM method combined with the deposition of a traveling head causes an amount of strain to be applied on the molten state chains during printing. A portion of this imposed strain, called printing-induced pre-strain, can be fixed and saved in the “frozen state” regarding the printing temperature, printing speed, cooling, and solidification rate [24,25,26,27]. In other words, the printed sample has a stored printing-induced pre-strain value, and the final distorted shape after the pre-strain relief should be considered the genuine temporary shape, while the printed shape is the temporary geometry of the in-printing programmed filaments with an amount of frozen pre-strain. The amount of pre-strain stored in the different layers can be directly related to the cooling rate, and the cooling rate is affected by the printing parameters [19,22,28]. This method uses the functionally graded pre-strain linearly along with the thickness of the samples printed with FDM, which is applied during extrusion, and, according to the printing parameters and cooling rate, a tension toward the printing direction from the first layer to the last one, it is stored linearly in the direction of thickness [27,29]. The second method is shape-shifting, which is used as one of the main 4D printing methods [30]. A wide range of shape-shifting is observed in nature, and their types originate from the arrangement of the constituent elements and response to various stimuli. The most important feature is the combination of shape-shifting and surface functionalization operations on a two-dimensional structure and conversion into a three-dimensional structure [31]. The mechanisms used in shape-shifting are in the two general categories that can be accessed and applied in the FDM or other AM techniques processes, and a lot of research has been conducted on them [32]. For the first type, the intralayer shear stress development can easily be made by the previously mentioned printing-induced pre-strain relief of extruded rasters on top of a passive adjacent layer, such as a polymer/paper composite, in which the paper acts as the passive layer. In contrast, the printed rasters respond (contract) to an increasing temperature. Thus, the planar=printed structure folds toward the active layer to form a 3D structure. The thermal expansion mismatch between the polymer and another passive material, like paper, upon heating can lead to a reversible shape transformation of the 3D curved structure to make it planar again. The second type does not involve the pre-strain relief for the earlier triggering while it works with just reversible shear stress development. For instance, a combination of an active material with a high swelling ability and a rigid passive material can cause a considerable shear stress mismatch by placing it in water, resulting in a drastic shape change with a great variety of possible mode shape regarding the printing strategy, making them highly applicable as origami. The transformed shape can turn back to its original shape by draining the structure [26,30,33,34].

These methods do not use SMPs in particular, and this has reduced costs, as well as a feed-stock limitation, and is very efficient for some special applications, such as sensors and actuators. However, limitations in programming and the degree of freedom to have an alterable temporary shape have weakened their performance such that it makes them incomparable as classic shape-memory structures by their confined performance [30]. Another limitation in these structures is the inability to repeat the shape memory cycle. In this research, for the first time, a layered structure consisting of two materials was printed to obtain a shape memory effect. The functions of the hard and soft phases in an SMP microstructure, which play essential roles in exhibiting an SME, were found in each printed layer. An elastomeric layer, with its strong elastic property, is responsible for maintaining the force with limited stress relaxation by its net points and guarantees that the original shape is recoverable. The other layer is a thermoplastic with hard-to-soft transitions of glass to rubber or melting ($T_{sw}$), which is the basis of the shape memory cycle. This layer acts as the switching phase and plays two different roles above and below the $T_{sw}$. At the $T\;<\;T_{sw}$, the high modulus and constraining effect prevent the return of the elastic layer, stabilize the temporary shape, and store the elastic force within the elastomeric layer, while in the case of the rubbery or molten layer ($T\;>\;T_{sw}$), the thermoplastic layer provides the essential softness for the relief of the elastic stress through shape recovery by a sharp drop in the elastic modulus. TPU was used for the elastic layer, and PCL and ABS were used for the thermoplastic layer in two ABS–TPU and PCL–TPU layered structures. The structures were printed by an FDM printer equipped with two nozzles, the shape memory test was repeated in a bending mode for five cycles, and the shape memory properties were extracted.

## 2. Materials and Method

In this research, ABS, PCL, and TPU were used as three common and high-consumption thermoplastic filaments in 3D printing with FDM. Commercial ABS granules were purchased from Baspar Chemi Sepidan Co., Ltd. (Tehran, Iran). Also, polyester-based TPU granules with the grade of 365A were prepared from Xiamen Keyuan Plastic Co., Ltd. (Xiamen, China). Commercial ABS granules were blended with 20 wt% polyester-based TPU by a melt-mixing method due to the increase in formability and toughness. The ABS-20TPU filament was prepared as one of the raw materials. Black-colored TPU and PCL filaments were provided by eSUN.

### 2.1. 3D Printing and SEM

ABS–TPU and PCL–TPU two-layer composite structures were printed to evaluate the shape memory effect. ABS and PCL were used as the plastic segment, and TPU played the elastomer segment role in both two-layered ABS–TPU and PCL–TPU printed structures. The hand-maker two-nozzle FDM 3D printer was used to prepare the beam specimens with dimensions of 50 mm × 10 mm × 3 mm. The thickness of both elastic and plastic layers was considered the same, and the total thickness of the samples was 3 mm. The printing conditions for all three materials were chosen according to Table 1. The parameters of the raster angle, layer thickness, and bed temperature were considered constant. The layer thickness and bed temperature were selected at 200 μm and 50 °C, respectively.

### 2.2. DMTA and SEM

Dynamic mechanical thermal analysis (DMTA) was performed to evaluate the thermomechanical behavior and determine the different thermal zones of PCL, ABS, and TPU. This test was performed by a dynamic mechanical thermal analyzer (Mettler Toledo, Switzerland) over a temperature range of −20 °C to 120 °C with a 5 °C/min heating rate and a constant 1 Hz frequency, using a cantilever beam printed in the geometry of 40 mm × 10 mm × 1 mm under bending mode (ASTM D4065-01 standard). Imaging was also obtained using an SEM to investigate the bond quality between the thermoplastics and TPU layers. Before imaging, the samples were broken down into liquid nitrogen and then coated with gold. Imaging was performed using a PhilipsXL30 SEM (Amsterdam, The Netherlands) with secondary electron imaging mode.

### 2.3. Shape Memory Cycle

**Figure 1:**
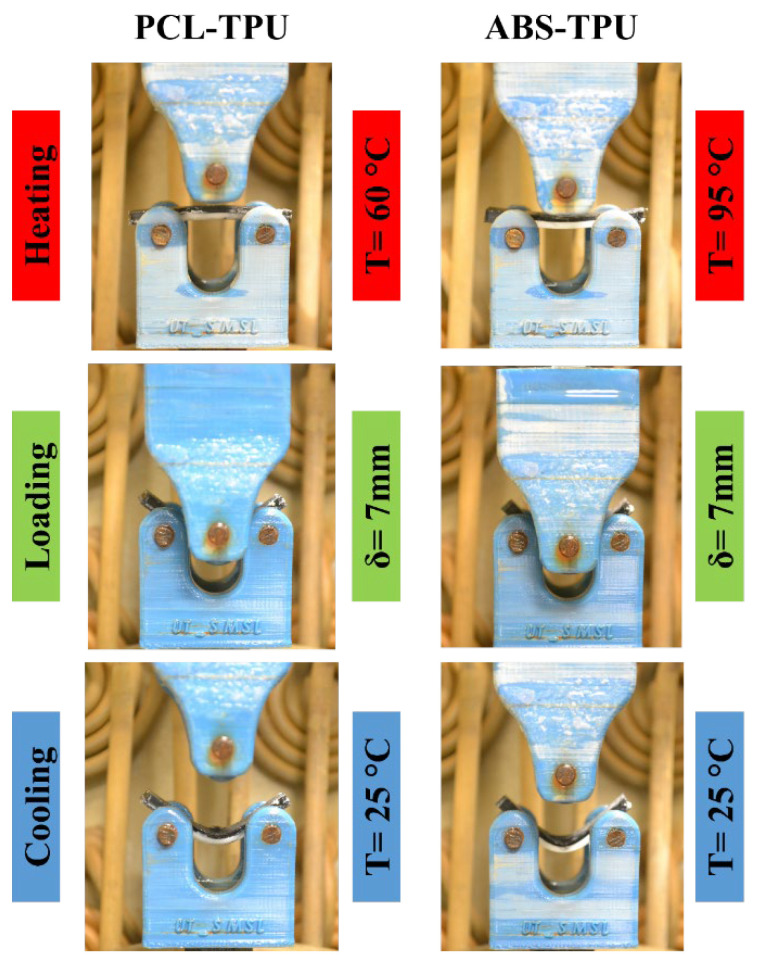
Steps and conditions of programming for two ABS–TPU and PCL–TPU composite structures.

A thermomechanical test was performed to evaluate the shape memory properties in flexural mode by applying a displacement of 7 mm at a displacement rate of 3 mm at PCL and ABS transition temperatures using a customized universal tensile testing machine containing a fluid chamber, designed and manufactured by the Khallagh Sanat Atieh Peyman company (Tehran, Iran). The steps and conditions of programming, such as heating, loading, and cooling for two ABS–TPU and PCL–TPU composite structures, are presented in Figure 1. The heating and cooling rates were set at 15 °C/min. The transition temperature was set at 60 °C and 95 °C for PCL and ABS, respectively. The shape memory cycle lasted up to 5 cycles for both the ABS–TPU and PCL–TPU structures. The shape memory cycle was applied as follows:

1. The chamber temperature was increased to the hard layer transition temperature;
2. The temperature was held for 240 s to heat the whole sample at the transition temperature and then deformation (7 mm) was applied;
3. The sample was cooled to a temperature of 25 °C with a cooling rate of 15 °C per minute;
4. The temperature was increased to the initial transition temperature and held for 240 s;
5. The amount of fixed and recovered deformation was measured.

## 3. Results and Discussion

### 3.1. DMTA

**Figure 2:**
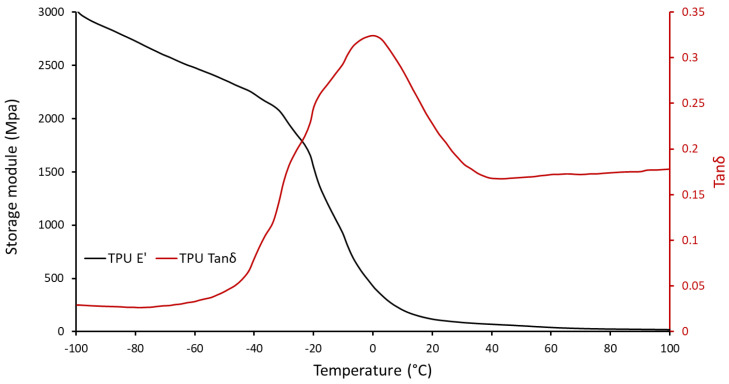
Storage module and tanδ of TPU.

**Figure 3:**
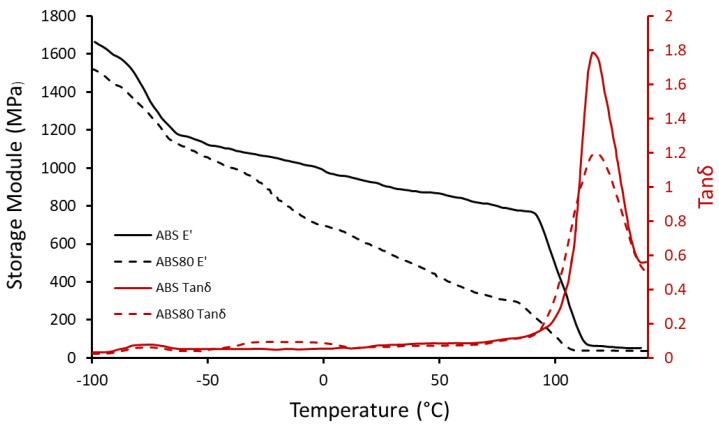
Storage module and tanδ of ABS and ABS80.

**Figure 4:**
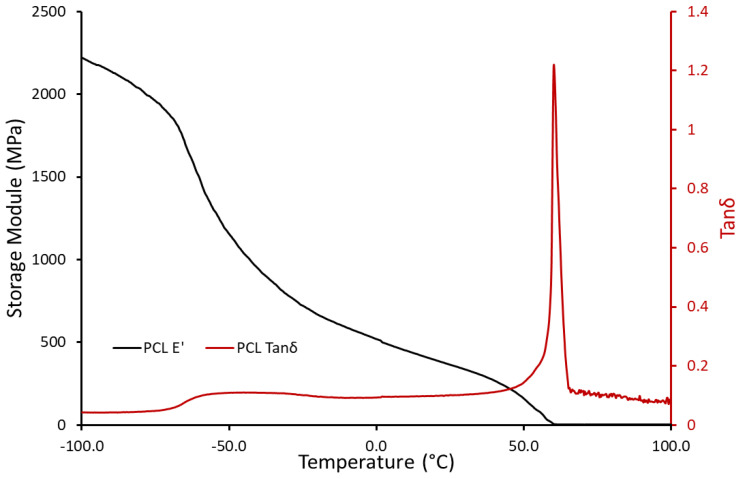
Storage module and tanδ of PCL.

The DMTA results for the raw materials are presented in Figure 2, Figure 3 and Figure 4. According to the DMTA curves of TPU (Figure 2), the glass transition phase started at −40 °C and continued until 25 °C. The highest point in the tanδ curve represents the glass transition temperature (−1 °C). The α transition temperature was −12 °C, which is the middle point of the storage module drop. Also, the storage module decreased from 80 MPa at room temperature to 25 MPa at 85 °C, and this decreasing trend continued to 19 MPa at 100 °C [35]. The scant rise in the storage module of TPU during the cooling process in the shape memory cycle slightly increased the stored stress in the elastomer. However, it cannot deteriorate the shape fixity, especially in the case of materials with high storage modules at room temperature, like ABS.

As it is illustrated in Figure 3, ABS had a glass transition range from 95 °C to 135 °C with a peak in the tanδ curve at 118 °C (T_g_). The storage module drop’s middle point of ABS occurred at 103 °C (Tα), and the storage module reduced from 760 MPa to 55 MPa during this transition. Moreover, between 0 °C and 85 °C, the storage module slightly decreased from 995 MPa to 780 MPa. Also, there was a β transition for ABS between −85 °C and −62 °C [35]. De León et al. [36] investigated the compatibility of TPU and ABS with Fourier transform infrared spectroscopy analysis in attenuated total reflectance mode, atomic force microscopy, and Raman microscopy. Also, the compatibility can be seen in the DMTA result (Figure 3) of ABS80 in this work. First, the glass transition range of ABS80 was from 89 °C to 130 °C. Second, the storage module of ABS 80 was lower than ABS in all of the temperature ranges. Third, the reduction rate of the storage module in the β transition of ABS was slightly decreased. Furthermore, there was an additional drop from around −40 °C to 0 °C, which is related to the glass transition range of TPU. Additionally, there was a significant continuous decreasing slope in the storage module curve of ABS80, which was from 695 MPa to 270 MPa between 0 °C and 85 °C, due to the incorporation of TPU in the blend. Also, the glass and α transition temperatures fell to 114 °C and 95 °C, respectively, and after surpassing the α transition temperature, the storage module dropped from 220 MPa to 32 MPa. As described above, all of these transitions are obvious in the tanδ curve. The basic concept of shape memory is based on a storage module of hard material above the switching temperature, which is the α transition temperature of ABS80. The recovery occurs at 100 °C, where the storage module of ABS80 is 88 MPa. This notable softening of ABS80 at this temperature can lead to the convenient release of stored stress in the black TPU layer during the recovery procedure.

Figure 4 depicts the DMTA curves of PCL, which represent the glass transition range and the melting point of this material. The glass transition range started at −65 °C and ended at −30 °C. In addition, the melting point is located at 60 °C. The storage module of this material reduced from 1175 MPa to 520 MPa in the temperature range between −50 °C and 0 °C, and this trend continued up to 160 MPa at 50 °C. However, after the melting procedure, the storage module dropped to 2 MPa [37]. This neglectable module, which is lower than TPU in temperatures higher than 60 °C, leads to a significant recovery ratio in a PCL/TPU structure for even cyclic shape memory performance.

### 3.2. SME and Description of the New Strategy

**Figure 5:**
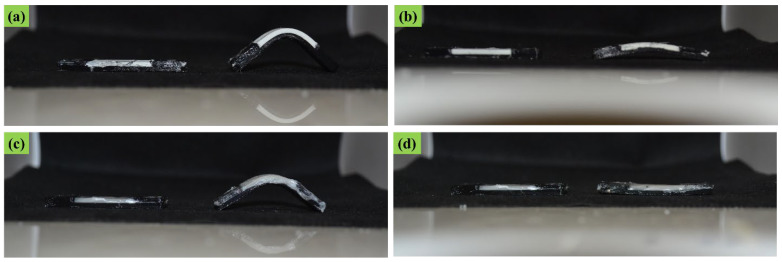
Shape memory properties in the first shape memory cycle: (**a**) fixity and (**b**) recovery for the ABS–TPU, and (**c**) fixity and (**d**) recovery for the PCL–TPU.

**Figure 6:**
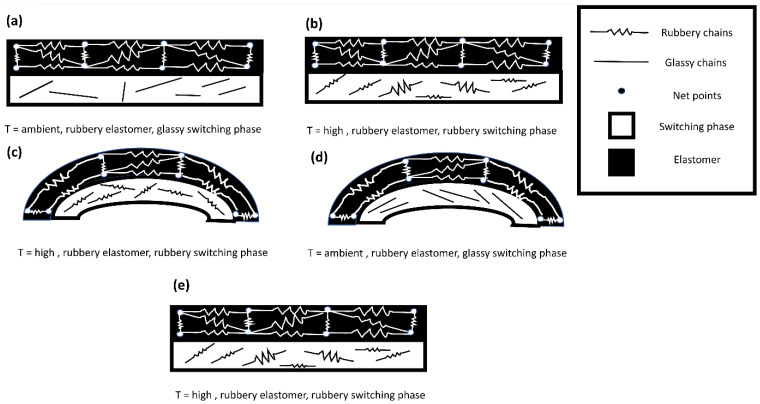
Schematic of samples during the shape memory cycle: (**a**) as-printed condition, (**b**) after heating, (**c**) after deformation, (**d**) after cooling, (**e**) recovery step.

**Figure 7:**
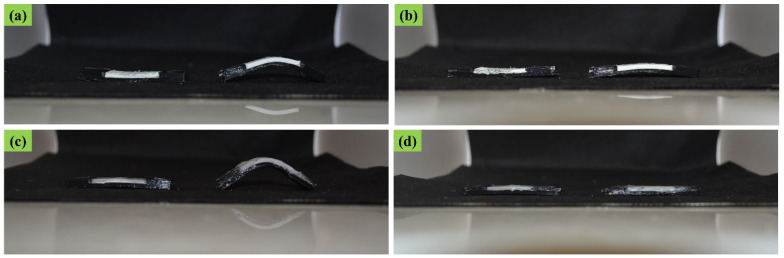
Shape memory properties in the last shape memory cycle: (**a**) fixity and (**b**) recovery for the ABS–TPU composite, and (**c**) fixity and (**d**) recovery for the PCL–TPU composite.

**Figure 8:**
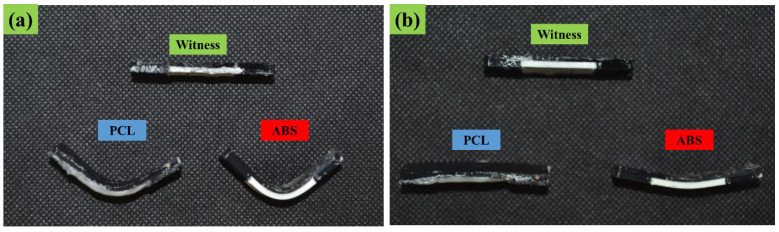
Visual comparison of shape memory properties for two structures of ABS–TPU and PCL–TPU composite structures: (**a**) shape fixity and (**b**) shape recovery.

In Figure 5, the programmed and recovered samples are seen next to the witness (as printed), after the first shape memory cycle, for both ABS–TPU and PCL–TPU composite structures under bending loading mode. The shape memory performance was extracted from Figure 5. The shape fixity and recovery ratios in the first cycle for ABS–TPU were 90.24% and 93.11%, respectively, which is very acceptable as a new method. These values for PCL–TPU are 77.42% and 100%, respectively, which indicates that this method is not dependent on the material and programming condition. In this strategy, the elastomer (TPU) and thermoplastic layers (ABS and PCL), which form a two-layer structure, are the same soft and hard phases in the classic shape memory materials, and each layer plays a role in each stage of the shape memory cycle, which is described in detail below. According to Figure 6, which is a schematic of the proposed method, in the heating step, increasing the temperature above the thermoplastic layer transition temperature, which can be the glassy transition temperature or melt temperature for amorphous (ABS) and semi-crystal thermoplastic (PCL), the state of the thermoplastic layer transferred from glassy to rubbery or melt states, which can be deformed even for rigid thermoplastics such as ABS. After applying the deformation to the two-layer structure, the deformation force is formed in two layers. In the cooling step, the elastic and thermoplastic layers show two completely different behaviors due to their nature. Due to the constraints created by the thermoplastic layer (higher elastic modulus), it is impossible to release the stored force and for the elastic layer to return to its original shape. It acts as the driving force of recovery in the final stage. Therefore, the elastomer layer is responsible for restoring the original shape. The thermoplastic layer also plays the role of the switching phase and has two different roles in higher and lower transition temperatures by a sharp change of the elastic modulus. In fact, in the cooling step, it plays a key role in stabilizing the temporary shape by creating a constraint, and in the recovery step, by decreasing the elastic modulus, it allows the force stored in the elastomer layer to be released, and the original shape is restored. In other words, changing the behavior of the thermoplastic layer in the rubbery and glassy region causes the formation of the shape memory effect in two-layer structures, and thermoplastic and elastomer layers play the role of energy storage and constraint, and are responsible for stabilizing the temporary and permanent shape, respectively. In fact, the choice of ABS and PCL with two different transition temperatures in the programming step (glassy transition and melting temperature) is due to studying this idea more comprehensively. Shape fixity and recovery above 90% for ABS–TPU demonstrate the efficiency of this method for achieving 4D structures. The possibility of loading in different modes and changing the desired shapes are other advantages of this structure. Also, in order to investigate the cyclic behavior of the two structures, the SME was repeated for up to five cycles. The results of shape fixity and recovery rations in terms of the number of shape memory cycles are presented in Table 2. The fixed and recovered samples after the fifth shape memory cycle for both ABS–TPU and PCL–TPU structures are presented in Figure 7. According to the results, a downward trend in shape fixity and shape recovery for both ABS–TPU and PCL–TPU structures are observed in the early shape memory cycles, and for the fourth and fifth cycles, the values are almost constant. Based on Figure 8, a visual comparison of shape memory properties for two structures of ABS–TPU and PCL–TPU in the last cycle proves that the PCL–TPU shows better cyclic shape memory behavior. A further drop in the elasticity modulus at the melting temperature for the PCL results in greater freedom of operation for the TPU to recover the permanent shape. On the other hand, the lower elasticity modulus of PCL compared to ABS at room temperature shows weaker resistance and constraint than ABS, and the amount of shape fixity for PCL is lower. However, this difference decreases in the final cycles, and the same shape fixity is obtained for both structures.

To achieve 4D printing, SMPs are usually used, and considering that this feature is not generally one of the inherent properties of them, it is necessary to blend and synthesize to achieve the SME. Therefore, the use of this operation requires the design and processing of smart polymer, which is a complex and costly task in most cases. In addition, another limitation is the printability of the processed polymers. In this study, a new strategy and design for achieving an SME and 4D printed two-layer composite structures is unveiled, thanks to FDM biomaterial printing of commercial filaments, which do not have an SME. Also, the use of commercial filaments reduces costs and provides ideal printing quality, and does not require additional operations. In the introduced method, because the recovery is performed by the TPU layers, and due to their favorable stress relaxation, these structures show better shape memory performance than thermoplastic SMPs. One of the main weaknesses of this class of smart materials is relaxation at the recovery temperature due to the lack of strong net points, such as chemical crosslinks or high crystallinity percentage. In short, according to the advantages provided, this method can be used in medical, aerospace, sensor, actuator, and electronic applications.

### 3.3. Microstructure and Interfacial Adhesion

Adhesion of two different materials is the key term for multi-material and composite printability. Thus, for success in multi-material composite printing, several notes should be considered to obtain good intralayer adhesion between two different polymers. An acceptable adhesion demands three important conditions as follows [38,39,40]:

- Good intermolecular interaction;
- Mechanical interlocking;
- Diffusion phenomenon.

The synergic combination of the aforementioned phenomenon leads to a promising interfacial adhesion, resultant shape memory behavior, and part integrity. The most determinative condition is intermolecular interaction, which shows the tendency of the two different polymers for microscopical mixing with each other. In fact, the strength of intermolecular interaction is named as compatibility between two polymers in the case of polymer blending, which reaches miscibility in the most strong intermolecular bonding [38,41]. The intermolecular bonding depends on the polarity of each polymer, which increases by containing polar groups or possible hydrogen bonding. For recognizing the pair of polymers with close polarity and good adhesion, solubility parameters or their blending condition (compatibility or miscibility) seems to be the best measure. The polar interactions can take place more efficiently by increasing the contact area, which is affected by intermolecular force and polymer rheological properties [40]. The other mentioned conditions have an auxiliary role in improving the interfacial bonding strength. Mechanical interlocking can be caused by key-hole tightening locking based on the surficial condition and possible roughness for this type of bonding, which can be found in high numbers in FDM 3D printing. The diffusion phenomenon can significantly strengthen the interfacial bonding by making a thin, narrow miscible alloy of the two polymers, providing integrity in the printed sample. Diffusion is mainly associated with temperature and time, as well as molecular weight, which determines the dynamic of a polymer chain to cross the interface [39,41]. All the mentioned parameters should be considered for justifying the interfacial condition of a polymeric multi-material part.

**Figure 9:**
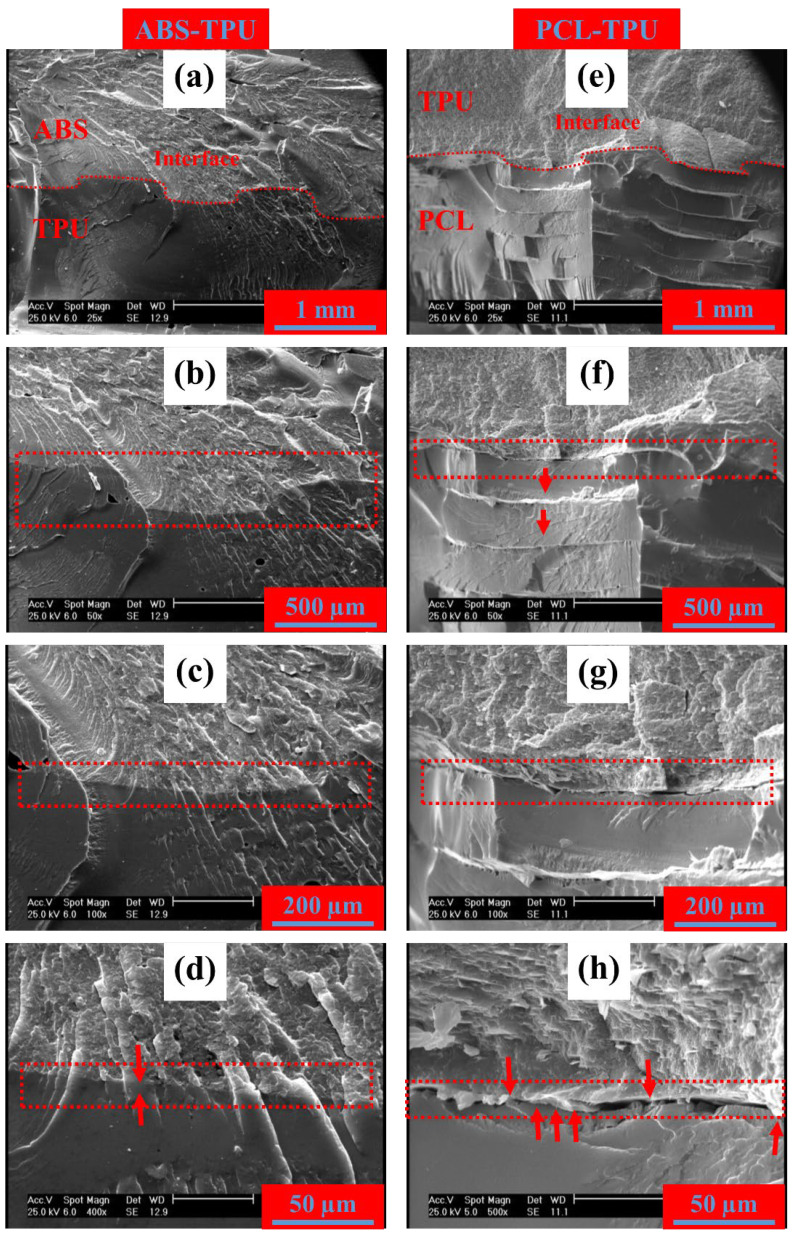
SEM photographs of ABS+TPU/TPU (**a**–**d**) and PCL/TPU (**e**–**h**) two-layer composite structures printed samples’ interfaces with magnifications of 5×, 50×, 100×, and 400×/500×, respectively.

Figure 9 shows the SEM photographs of the interface between the TPU elastomer and the rigid layer of the ABS/TPU blend and PCL at the right and left sides of the image, respectively. According to Figure 9a–d, the interface (red-dashed line) is easily detectable, which separates each photograph into two sections of lighter and darker portions with different fracture morphologies. In a wider view, the interface is not perfectly linear and has repeatable S shape zigzag geometry, which expands the interfacial area for stronger possible polar bonding and provides the possibility of mechanical interlocking, as well [40]. The magnified photographs do not show any local segregation in the interface between ABS/TPU and TPU. As shown in Figure 9d, the cracks crossed the interface, and even their growth direction was kept unchanged. It shows the multi-material 3D printed part’s integrity as a result of superior interfacial bonding and possible interfacial molecular diffusion of the ABS/TPU and TPU layers [39,41]. A 20 wt.% of TPU in ABS may act efficiently on diffusion and make an excellent interfacial bonding with TPU. In addition, probable hydrogen bonding between highly polar groups of TPU and acrylonitrile and aromatic moieties of ABS may be another reason for this excellent interfacial bonding [36]. Conclusively, the SEM photographs illustrated superior interfacial bonding and part integrity in the case of multi-material FDM 3D printed ABS/TPU and TPU, which guarantees its functionality under loading and shear stress mismatches during the SME. The compatibility of these materials has also been confirmed in previous research. Harris et al. [42] investigated the interfacial adhesion on a three-layered ABS/TPU/ABS printed structure. The shear test proved that bonding between TPU and ABS was comparable to commercial adhesive. De León et al. [36] studied the compatibility of the ABS and TPU blends. The results demonstrated a homogeneous distribution of ABS and TPU in the blends due to their superior compatibility. The interface of PCL and TPU (see Figure 9d) have the same geometry as the ABS, while a local incomplete intralayer coalescence can be observed in the PCL section. As shown in Figure 9e, each single printed PCL raster is detectable separately (red arrows). Microsegregations in the interface are seen in some areas, but there is good bonding in the interface, especially on the right side. The relatively high hardness of the PCL and its pasty melt may lead to incomplete interfacial diffusion [43]. Connections have remained between the two layers, even in the most segregated area (red arrows in Figure 9h). However, it seems that the brittle fracture-induced cracks proceed with their growth inside the interface, resulting in detectable segregations in the SEM micrographs. In practice, the PCL–TPU bilayer samples did not show any interfacial weakness or failure during the SME cycle. However, the SEM photographs show that ABS/TPU can form a more effective interfacial bonding than PCL, such that pre-imaging brittle fracture cannot cause even microsegregations due to its possible diffusion, while PCL can form a good functional bonding by its polarity without a considerable diffusion, resulting in microsegregations upon brittle cross-sectional fracture. Several studies are available in PCL/TPU blends and coextrusion. Zheng et al. [44] produced a multilayered coextruded structure of PCL and TPU to obtain an SME. The PCL played the transition role by its melting/crystallization, while the TPU provided the essential elasticity for recovery. The layer bonding in the coextruded sample was high and their blends showed an immiscible morphology with high compatibility, which demonstrates the good intermolecular interaction between the two components. Bhattacharya et al. [43] prepared a shape memory-assisted self-healing blend of PCL/TPU. The morphology was immiscible with high compatibility because of their excellent intermolecular interaction. The good compatibility of PCL and TPU occurs in polyester-based TPU. The compatibility between PCL and TPU reaches its maximum, which is called molecular miscibility, by employing the TPU containing PCL-based soft segment [45,46].

## 4. Conclusions

In this study, for the first time, a new technique was used to create 4D printed composite structures with excellent shape memory performance without the use of smart materials by printing two-layer composite structures consisting of commercial filaments with FDM. For this purpose, the ABS, PCL, and TPU filaments available on the market were used, and the following outstanding results were obtained:

- ABS and PCL in two-layer composite structures play the role of the switching phase by changing the state and behavior at higher and lower transition temperatures, and the elastic behavior of TPU also helps to complete the shape memory cycle by storing and recovering force during loading and recovery stages, respectively;
- Both ABS–TPU and PCL–TPU composite structures had a suitable shape memory performance. However, the ABS–TPU and PCL–TPU composites had more shape fixity and recovery ratio, respectively. In the first cycle, ABS–TPU and PCL–TPU had 100% shape fixity and recovery ratios, respectively;
- The cyclic behavior of both two-layer composite structures showed that shape memory performance decreased with increasing shape memory cycle and was almost constant in the final cycles. The PCL–TPU composite also showed better cyclic behavior, especially in shape recovery. This method may have many applications in bio, sensor, and actuator fields due to the minimization of stress relaxation, which is the main weakness of thermoplastic shape memory polymers;
- The SEM photographs illustrated superior interfacial bonding and part integrity in the case of multi-material FDM 3D printed ABS/TPU and TPU, which guarantees its functionality under loading and shear stress mismatches during the shape memory effect. The interface of PCL and TPU had the same geometry as the ABS, while a local incomplete intralayer coalescence was observed in the PCL section.

## Figures and Tables

**Table 1:** Variable parameters for printing ABS, PCL, and TPU.

Printing Parameters	ABS	PCL	TPU
Velocity (mm/s)	50	15	15
Temperature (°C)	240	100	240
Nozzle diameter (mm)	0.8	0.8	0.4

**Table 2:** Results of cyclic shape memory effect.

Two-Layer Composite Structures	Cycle	Shape Fixity Ratio (%)	Shape Recovery Ratio (%)
ABS–TPU	1	90.2 ± 0.8	93.1 ± 1.3
	2	85.3 ± 1.0	82.6 ± 1.2
	3	79.8 ± 0.4	72.7 ± 0.7
	4	68.3 ± 0.4	69.8 ± 0.5
	5	66.3 ± 0.2	69.3 ± 0.1
PCL–TPU	1	77.4 ± 1.6	100 ± 0.0
	2	72.7 ± 1.2	100 ± 0.0
	3	69.3 ± 1.3	98.3 ± 0.1
	4	68.4 ± 1.0	97.2 ± 0.2
	5	68.2 ± 0.8	97.0 ± 0.2

## Data Availability

All data are included in the article.
